# Modeling Nociception in Zebrafish: A Way Forward for Unbiased Analgesic Discovery

**DOI:** 10.1371/journal.pone.0116766

**Published:** 2015-01-14

**Authors:** Andrew Curtright, Micaela Rosser, Shamii Goh, Bailey Keown, Erinn Wagner, Jasmine Sharifi, David W. Raible, Ajay Dhaka

**Affiliations:** 1 Department of Biological Structure, University of Washington, Seattle, Washington, 98195, United States of America; 2 Neurobiology and Behavior Graduate Program, University of Washington, Seattle, Washington, 98195, United States of America; University of South California, UNITED STATES

## Abstract

Acute and chronic pain conditions are often debilitating, inflicting severe physiological, emotional and economic costs and affect a large percentage of the global population. However, the development of therapeutic analgesic agents based primarily on targeted drug development has been largely ineffective. An alternative approach to analgesic development would be to develop low cost, high throughput, untargeted animal based behavioral screens that model complex nociceptive behaviors in which to screen for analgesic compounds. Here we describe the development of a behavioral based assay in zebrafish larvae that is effective in identifying small molecule compounds with analgesic properties. In a place aversion assay, which likely utilizes supraspinal neuronal circuitry, individually arrayed zebrafish larvae show temperature-dependent aversion to increasing and decreasing temperatures deviating from rearing temperature. Modeling thermal hyperalgesia, the addition of the noxious inflammatory compound and TRPA1 agonist allyl isothiocyanate sensitized heat aversion and reversed cool aversion leading larvae to avoid rearing temperature in favor of otherwise acutely aversive cooler temperatures. We show that small molecules with known analgesic properties are able to inhibit acute and/or sensitized temperature aversion.

## Introduction

The ability to sense pain is normally advantageous, motivating animals to withdraw from injurious stimuli, to protect damaged tissue allowing it to heal, and to avoid dangerous environments in the future. However, acute and chronic pain affects hundreds of millions of people and imposes a severe emotional and economic burden on both individuals and society as a whole. Pain is a major symptom in many illnesses, and can also be particularly debilitating when it becomes disassociated from injury or illness, entering a chronic phase in which pain itself becomes the disease [1]. Despite massive investment in the past half century, there has been limited success in the development of novel analgesic compounds. The dominant drug development model follows a preclinical path in which a selected target is subjugated to high-throughput *in vitro* screens, the generation of lead compounds, and testing in disease model systems for safety and efficacy assessments. While this approach can be successful, it often leads to failure due to poor target selection, the inability to model complex behaviors using *in vitro* testing and/or ineffectiveness and unforeseen side effects in animal model testing [2]. In fact many current drugs such as NSAIDS, gabapentin and ketamine were identified due to their analgesic properties prior to the identification of their targets. While may not be possible to measure pain due to its complexity in animal model systems, measurement of nociception, the behavioral response to noxious stimuli, is routinely used to measure the efficacy of potentially analgesic compounds [3, 4]. It could therefore be advantageous to develop high-throughput phenotypic assays to model complex nociceptive behaviors in which one could screen for novel analgesics in an unbiased manner. While much work has been performed using rodents to model nociceptive behavior, screening for small molecule analgesics using these models is neither practical nor feasible due to the high cost, time and manpower necessary to conduct such a screen.

Zebrafish have a number of attributes that lend themselves to inquiries into the biology of nociception. They can be generated in large numbers, have relatively low maintenance costs, develop very quickly such that embryos/larvae have an intact nervous system that allows them to perceive and respond robustly to sensory stimuli and they have been successfully utilized in a number of behavioral screens [5–11]. More importantly, a growing body of evidence suggests that the development and organization of both peripheral and central nociceptive processing systems is similar between teleost fish such as the zebrafish and other vertebrates such as mammals even at early larval stages [12–14].

Here we report the development of a novel behavioral assay to model acute and sensitized temperature aversion with large numbers of zebrafish larvae. Furthermore, we show that these assays can be used to screen for small molecule analgesics, by showing that previously described analgesics with different pharmacological properties are able to suppress noxious avoidance behavior.

## Materials and Methods

### Zebrafish

Zebrafish were maintained at 28.5°C on a 14 h/10 h light/dark cycle following established methods and all experiments were conducted with the approval of the University of Washington Office of Animal Welfare Institutional Animal Care and Use Committee. Larvae were maintained in an E2 medium (EM) and staged as described previously (Kimmel et al., 1995). All experiments utilized 30–90 larvae per assay, at stages before sex could be determined.

### Drug Preparation

Clonidine hydrochloride (C7897; Sigma), Amitriptyline hydrochloride (A8404; Sigma), HC-30031 (TRPA1 antagonist; H4415; Sigma), Gabapentin (G154; Sigma), A-803467 (Nav1.8 blocker; 2976; Tocris), TC-N1752 (Nav1.7 blocker; 4435; Tocris), Atenolol (A7655; Sigma), Ibuprofen (I4883;Sigma), Naloxone (0599; R&D Systems) Atenolol (A7655; Sigma), Lamotrigine (L3791; Sigma), Enalipril (CDS020548; Aldrich), Ethyl 3-aminobenzoate methanesulfonate (Tricaine; E10521; Sigma), Omeprazole (O104; Sigma), Promethazine hydrochloride (P4651: Sigma), Diphenhydramine hydrochloride (D3630; Sigma), and Pancuronium bromide (P1918; Sigma) were dissolved in DMSO to a 1000x concentration. Buprenorphine hydrochloride (12496–0757; Reckitt Benckiser Pharmaceuticals) was obtained as a 3mg/mL injection solution from UW Medicine Drug Services. Candidate analgesics were further diluted in a solution of EM and 2% DMSO for the behavioral assay.

### Temperature Aversion Assay

The Temperature Aversion apparatus consists of three elements: a dual solid-state heat/cool plate (AHP-1200°CP; Teca); a Canon high-definition digital video camcorder; and custom-made choice testing arenas. Oval shaped arenas were machined out of 3mm thick silicone rubber to final dimensions of 20mm by 8mm. Arenas were bonded to 0.05mm thick aluminum shim (40002; ShopAid) with a waterproof adhesive (00688; DAP).

Randomly selected five days post-fertilization (5dpf) zebrafish embryos were caught with a cut 200uL pipette tip and placed in 0.1ml EM in the previously described arenas. The arenas were then loaded with 0.1ml of candidate analgesic in 2% DMSO or a 2% DMSO vehicle control to achieve a final concentration of 1% DMSO and the reported concentration of analgesic. Embryos were then incubated at 28.5°C for 10 minutes. After incubation, the choice testing arenas were transported to the dual-sided heat/cool plate. One side of the heat/cool plate was maintained at a constant 28.5, while the temperature of the other side was adjusted according to the experiment. The choice testing arenas were centered on the heat/cool plate boundary such that each arena was half way on each side. Each trial consisted of 4 minutes of video recorded from a fixed position above the hot/cold plate.

### Sensitized Temperature Aversion Assay

The sensitized pain assay was carried out in a similar manner to the choice test assay. However embryos were caught in 50μL of EM then loaded into the arenas with 50uL of 2μM allyl isothiocyanate (AITC; 377430; Sigma) and 100μL of the prospective analgesic for a final concentration of 0.5μM AITC.

### Washout Assay

Zebrafish embryos were caught and placed in a mesh bottomed Corning netwell (CLS3521; Sigma) and incubated in 4uM AITC for 10 minutes. After incubation, embryos underwent 4 successive washes in EM and were loaded into the test arenas in 0.2ml EM. At selected time points, embryos were tested for temperature aversion at 24.5°C versus 28.5°C for 4 minutes.

### Data Analysis

Videos from each trial where cropped to 4 minutes and imported into EthoVision XT locomotion tracking software (Noldus Information Technology). A custom template defined two zones within each choice testing arena such that one corresponded to the 28.5°C side and the other to the experimental temperature. The position and velocity of each embryo was tracked for both zones and exported into Microsoft Excel. Both position and velocity data were normalized to their respective controls, either 1% DMSO or 0.5µM AITC, by dividing the mean position or velocity value by the result of a control experiment performed on the same day. Standard errors were propagated with the same linear transformation.

### Statistical Analysis

Position and velocity data were imported from Microsoft Excel into GraphPad Prism 6 statistical analysis software. Two-way ANOVA was performed on both position and velocity data, followed by post-hoc analysis using Sidak’s multiple comparison correction. When only a single condition was tested, such as when non-analgesic compounds were screened in the sensitized temperature aversion assay, the entire panel of drugs was treated as single experiment. Asterisks in all figures represent two-way ANOVA post-hoc comparisons unless otherwise noted.

## Results

### Acute Temperature Discrimination

**Figure 1:**
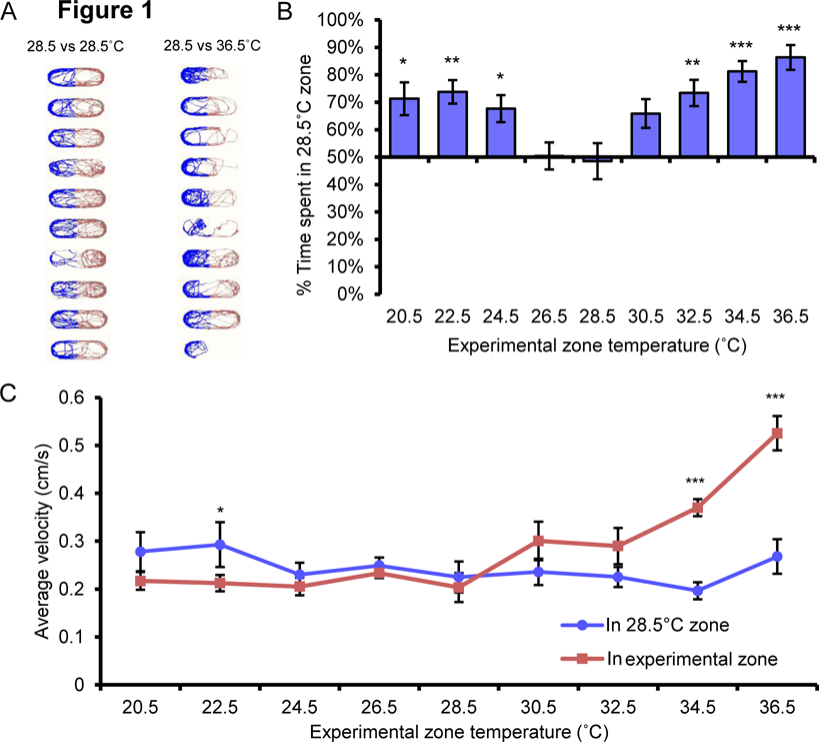
5 dpf zebrafish larvae robustly avoid hot and cold temperatures. **A**, Track plot of zebrafish larvae where the position of the larvae in the control zone is represented in blue and the position of the larvae in the experimental temperature zone are represented in red. Left, control zone = 28.5°C, experimental zone = 28.5C; right, control zone = 28.5°C, experimental zone = 36.5°C. Larvae show no choice when each zone is set to 28.5°C, but spend less time in the experimental zone when it is set to 36.5°C. **B**, Distribution of time larvae spend in the 28.5°C control zone when experimental zone temperature set as indicated. Animals showed no choice when control and experimental zones were set to 28.5°C but preferred the 28.5°C control zone when juxtaposed with hotter and colder temperatures. **C**, Velocity of larval movements in control and experimental zones. The velocity of larvae was greater in the 34.5°C and 36.5°C zones than in the corresponding 28.5°C zone. * p<0.05, ** p<0.01, *** p<0.001. Error bars represent SEM.

We have developed a two-temperature discrimination assay that records the preference of zebrafish larvae to rearing temperature versus an aversive testing temperature. A potential advantage of this assay for conducting analgesic screens is that higher order supraspinal pain pathways may be employed by the larvae to escape and avoid aversive temperatures versus simpler acute locomotor based assays, which may only require spinal based reflexive movement [3, 8, 15–17]. Thus it is possible that a choice based screen could identify analgesics that would go undetected in a reflexive behavior based assay. We previously reported that groups of freely swimming 5dpf zebrafish larvae robustly choose their rearing temperature 28.5°C over noxious hot 36.5°C and noxious cold 12°C temperatures (Gau et al., 2013). In order to remove the possibility that group behavior was influencing temperature avoidance, we designed an apparatus where individually arrayed larvae were allowed to freely explore an arena where two different temperatures could be set in each half of the arena (Fig. 1A). For each test, larvae chose between a control zone set at 28.5°C and an experimental zone set to hotter or colder temperatures. 5dpf zebrafish larvae consistently chose the 28.5°C control zone over the experimental zone set to temperatures greater than 30.5°C, with maximal avoidance at 36.5°C (Fig. 1B). Larvae also avoided the experimental zone when it was set to temperatures cooler than 24.5°C (Fig. 1B). Fish did not spend a statistically different amount of time in either zone when both were set to 28.5°C indicating that there were no internal biases in our apparatus.

We also measured the velocity of larval movement in control and experimental zones. As temperatures in the experimental zone got hotter the velocity of the larvae increased compared to the velocity of the same larvae in the control 28.5°C zone (Fig. 1C). These findings are in alignment with our previous finding that larval locomotion increased with temperature, and was similar to the increased locomotion observed in larvae exposed to noxious compounds such as AITC (Gau et al., 2013). Larvae showed little to no changes in velocity set to temperatures cooler than 28.5°C (Fig. 1C). It appears then that at lower temperatures, velocity or locomotion measurements may not be a good measure of acute temperature aversion compared to place aversion. In sum our findings suggest that zebrafish larvae have a fairly narrow range of preferred temperatures and temperatures that deviate from this range drive aversive behavior.

### Sensitized temperature discrimination

**Figure 2:**
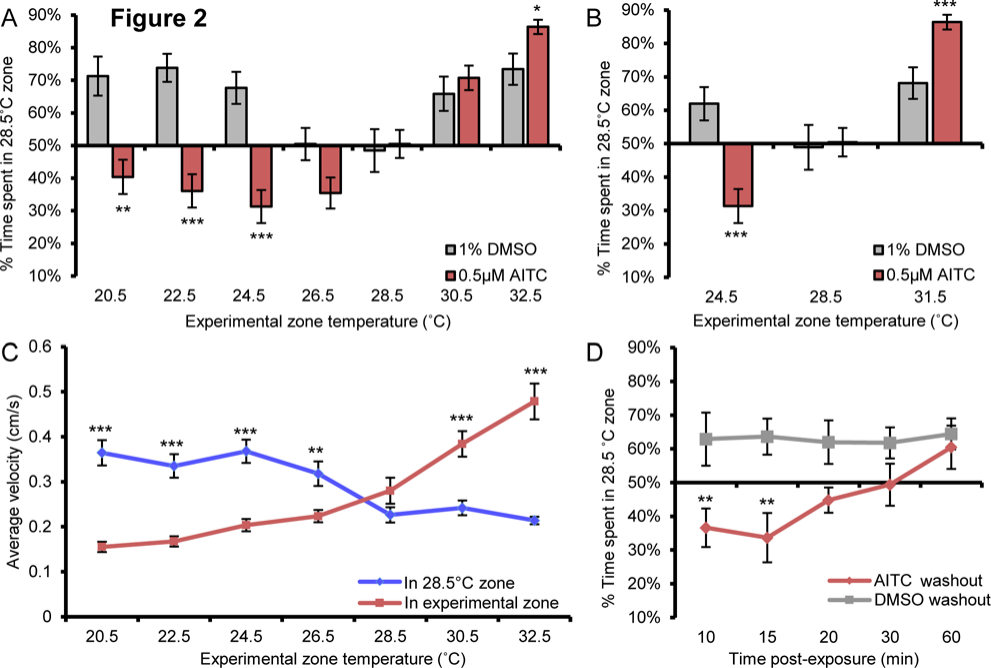
AITC promotes thermal aversion and reverses cool aversion. **A**, 5dpf larvae incubated in 0.5μM AITC showed increased aversion to 32.5°C and preferred temperatures below 24.5°C when compared to responses of vehicle-treated larvae when tested against 28.5°C. AITC treated larvae also showed aversion to 28.5°C when matched to temperatures between 26.5°C and 22.5°C when compared against chance. **B**, AITC increases aversion to 31.5°C and reverses aversion for 24.5°C when compared to vehicle treated larvae when tested against 28.5°C (2-way anova post hoc comparison) while not affecting zone preference in the 28.5°C versus 28.5°C test. **C**, AITC treated larvae display increased velocity above 30.5°C when compared to velocity in the 28.5°C zone (paired student’s t-test). Conversely, AITC treated larvae showed an increased velocity in the 28.5°C zone when compared to velocity in zones cooler than 26.5°C (paired student’s t-test). **D**, AITC dependent aversion of 28.5°C when tested against 24.5°C is time dependent. Up to 15 min after AITC washout, treated larvae avoided 28.5°C more than vehicle treated fish but not after 20 min after washout (2-way anova post hoc comparison). * p<0.05, ** p<0.01, *** p<0.001. Error bars represent SEM.

Inflammatory compounds such as mustard oil (AITC) can reduce the threshold of stimuli necessary to evoke a nociceptive response and are often used to invoke behavioral sensitization [18–20]. To model sensitized temperature aversion, zebrafish larvae (5dpf) were pre-incubated (10 min) with 0.5μM AITC and tested for temperature avoidance (Fig. 2). We found that incubation with 0.5μM AITC increased avoidance of temperatures greater than or equal to 31.5°C compared to vehicle treated larvae (Fig. 2A,B). More strikingly, AITC treatment caused the larvae to prefer normally neutral cooler (26.5°C) and aversive cooler (22.5–24.5°C) temperatures compared to 28.5°C (Fig. 2A). When AITC treated larvae were given a choice between 20.5°C and 28.5°C, the larvae showed no preference for either zone when compared against chance but did spend significantly less time at 28.5°C than vehicle treated larvae, which strongly prefer the 28.5°C zone (Fig. 2A). It seems that any preference for cool temperature over 28.5°C in the presence of AITC is lost due to the aversive nature of increasingly cold temperature (Fig. 1B). We also found that while AITC-treated larvae showed no changes in velocity of movement when both zones were at 28.5°C, they showed significant increases in velocity in a warmer zone at lower threshold (≥30.5°C) than for vehicle treated larvae (≥34.5°C; Fig. 2C compare to Fig. 1C). AITC-incubated larvae also showed increased velocity in the 28.5°C zone when tested against cooler temperatures (20.5–26.5°C; Fig. 2C). This was in contrast to our earlier observation where larvae had nearly indistinguishable velocity at 28.5°C versus cooler temperatures (Fig. 1C). These results support the interpretation that after AITC treatment, lower temperatures are less noxious than rearing temperature. Taken together these data suggest that velocity and temperature aversion is influenced by the relative level of noxious stimuli in each of the juxtapositioned testing environments.

The most common behavioral paradigms for measuring sensitized temperature hyperalgesia involve the single application of an inflammatory agent followed by the repeated measurement of temperature invoked behavior at regular time intervals until the heightened response to temperature stimuli resolves to baseline or vehicle control levels [20, 21]. These models are thought to mimic changes in nociception brought about by acute injuries such as those caused by surgery and other painful inflammatory conditions [20]. To test whether zebrafish show a similar alteration in hyperalgesic response, larvae were incubated in AITC (2μM) or vehicle control (1% DMSO) for 10 min followed by washout of AITC. We compared temperature aversion for 28.5°C versus 24.5°C since temperature preference was reversed after AITC treatment (Fig. 2A). While animals continued to prefer the cooler temperature immediately after AITC washout, by 30 min after washout they showed no preference and by 60 min they showed the same preference for the warmer temperature as vehicle-treated controls (Fig. 2D). This result suggests that our sensitized temperature discrimination assay may appropriately reflect sensitized nociception.

### Buprenorphine reverses acute and sensitized thermal aversion

**Figure 3:**
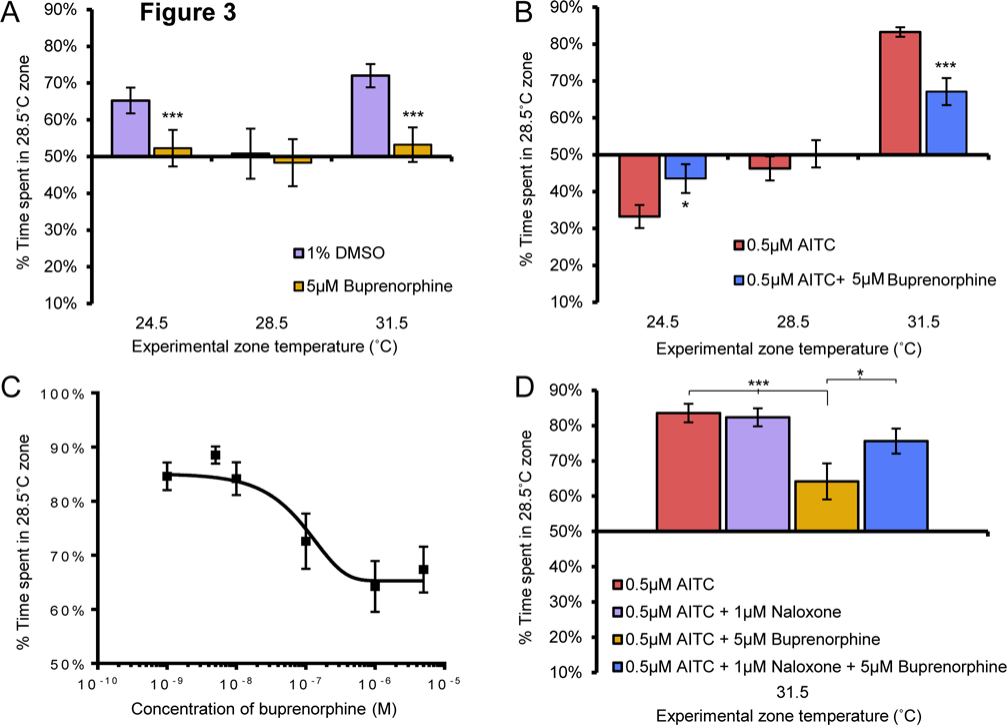
Buprenorphine temperature aversion in a dose-dependent and specific manner. **A**, Buprenorphine (5μM) significantly reduced 24.5°C and 31.5°C aversion when compared to vehicle treated larvae (2-way anova post hoc comparison) and eliminated aversion to 24.5°C and 31.5°C as measured by no significant difference from chance (1-way anova against a hypothetical mean of 50%). Buprenorphine did not induce aberrant zone preference (1-way anova against a hypothetical mean of 50%) and did not differ from vehicle treated larvae in the 28.5°C vs 28.5°C assay (2-way anova post hoc comparison). **B**, Buprenorphine significantly reduced AITC sensitized preference for 24.5°C and aversion for 31.5°C when compared to vehicle treated larvae (2-way anova post hoc comparison). Buprenorphine did not induce aberrant zone preference (1-way anova against a hypothetical mean of 50%) and did not differ from vehicle treated larvae in the AITC sensitized 28.5°C vs assay (2-way anova post hoc comparison). **C**, Buprenorphine inhibits AITC sensitized thermal aversion in a dose-dependent manner. **D**, Buprenorphine inhibition of AITC sensitized thermal aversion is reversed by addition of naloxone (2-way anova post hoc comparisons). * p<0.05, ** p<0.01, *** p<0.001. Error bars represent SEM.

**Figure 4:**
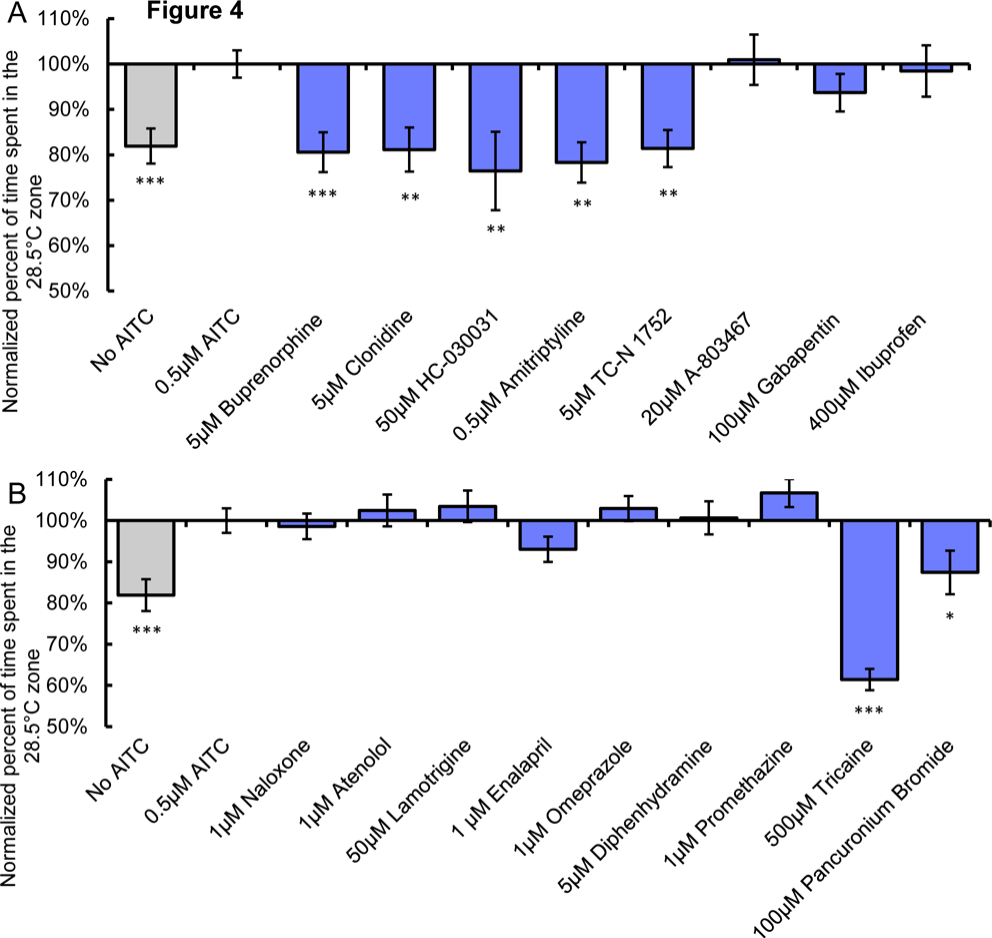
Analgesics can inhibit AITC sensitized thermal aversion. **A**, The percent time larvae incubated with analgesic plus AITC (0.5μM) spent in the 28.5°C zone during a 28.5°C vs 31.5°C assay normalized to percent time larvae incubated in AITC (0.5μM) + vehicle alone spent in the 28.5°C zone. No AITC larvae were treated with 1% DMSO vehicle alone. **B**, The percent time larvae incubated with non-analgesic plus AITC (0.5μM) spent in the 28.5°C zone during a 28.5°C vs 31.5°C assay normalized to percent time larvae incubated in AITC (0.5μM) + vehicle alone spent in the 28.5°C zone. No AITC larvae were treated with 1% DMSO vehicle alone. * represent statistical difference from AITC (0.5μM) alone condition (2-way anova post hoc comparison) * p<0.05, ** p<0.01, *** p<0.001. Error bars represent SEM.

If our temperature-based place preference assay reflects nociceptive behavior, then analgesic compounds should alter the ability of the zebrafish larvae to discriminate between innocuous and noxious environments. To test this concept, we investigated whether buprenorphine, a partial opioid agonist that is used in the treatment of acute and chronic pain, could suppress thermal discrimination in our assays [22–24]. We focused our analysis primarily on place aversion rather than velocity since this measurement was the most consistent across all testing conditions. Experimental temperature zones were set at 31.5°C or 24.5°C and compared to 28.5°C as these were the minimal temperatures that we observed place preference in the acute temperature discrimination assay and also revealed enhanced or reversed choice in the sensitized temperature discrimination assay. Buprenorphine completely inhibited both acute hot and cold temperature aversion (Fig. 3A). Furthermore, buprenorphine suppressed sensitized heat avoidance after AITC treatment, reducing choice to levels seen with larvae not treated with AITC, and eliminated AITC induced sensitized cool preference (Figs. 3A, 3B, 4A). We also noted that buprenorphine reduced velocity in the 31.5°C zone of AITC treated larvae to levels seen for larvae that were not exposed to AITC, suggesting that buprenorphine reversed the sensitizing effects of AITC but not did not effect locomotion (Table 1). To further investigate if buprenorphine was suppressing locomotion and therefore place preference, we measured the effects of buprenorphine on velocity of larvae incubated at 28.5°C alone and found no difference in velocity between vehicle treated and buprenorphine treated larvae (Table 2). This suggests that buprenorphine treatment likely effected temperature aversion and not locomotion. If buprenorphine were acting to reverse thermal aversion, we would also expect a correlation between its dose and the behavioral response. To test this we focused on the sensitized heat aversion assay as this behavior was consistently robust and may reflect inflammatory nociception. Indeed, we found that buprenorphine reversed thermal aversion in a dose dependent manner (Fig. 3C). Furthermore, the opioid receptor antagonist naloxone abolished the effects of buprenorphine on AITC induced sensitized thermal aversion indicating that buprenorphine was acting directly on opioid receptors to inhibit nociception. Naloxone plus buprenorphine treated animals did not differ significantly versus vehicle or naloxone only-treated larvae in the amount of time spent in the 28.5°C zone. On the other hand buprenorphine alone treated larvae spent significantly less time in the 28.5°C zone than vehicle, naloxone alone or naloxone plus buprenorphine treated larvae (Fig. 3D). These data support the conclusion that AITC sensitized thermal aversion is reflective of nociceptive behavior and that this assay could be useful in the identification of analgesic compounds.

**Table 1:** AITC Sensitized Velocity in 31.5°C Zone.

**Drug**	**Normalized Velocity**	**SEM**	**p-value**
0.5µM AITC	1.00	0.05	
No AITC	0.56	0.05	p<0.001
Analgesics			
5µM Buprenorphine	0.62	0.06	p<0.001
5µM Clonidine	0.52	0.07	p<0.001
50µM HC-030031	0.52	0.05	p<0.01
0.5µM Amitriptyline	0.64	0.04	p<0.05
5µM TC-N 1752	0.76	0.10	p>0.05
20µM A-803467	0.62	0.04	p<0.001
100µM Gabapentin	0.84	0.07	p>0.05
400µM Ibuprofen	0.79	0.05	p>0.05
Non-Analgesics			
1µM Naloxone	0.98	0.02	p>0.05
1µM Atenolol	0.46	0.05	p<0.001
50µM Lamotrigine	0.70	0.06	p<0.05
1µM Enalipril	0.89	0.08	p>0.05
1μM Omeprazole	1.01	0.08	p>0.05
5μM Diphenhydramine	1.00	0.10	p>0.05
1μM Promethazine	1.02	0.07	p>0.05
Anesthetic/Paralytic			
500µM Tricaine	0.02	0.00	p<0.001,p<0.001*
100μM Pancuronium Bromide	0.36	0.07	p<0.001,p<0.05*

Effect of small molecules on larval velocity in the 31.5°C during AITC sensitized thermal aversion 28.5°C assay. Small molecule analgesics (buprenorphine, clonidine, HC-030031, amitriptyline) inhibited AITC sensitized thermal aversion suppressed larval velocity significantly from AITC (0.5μM) + vehicle alone conditions but had velocity indistinguishable from No AITC controls. Other small molecules (A-803467, lamotrigine, enalipril) also inhibited larval velocity but did not effect sensitized thermal aversion possibly indicative of non-analgesic effects on locomotion. A third category of anesthetic/paralytics (tricaine, pancuronium bromide) suppressed sensitized thermal aversion and velocity, but suppressed velocity significantly below the velocity of No AITC controls possibly indicative of non-analgesic effects on locomotion. p-value, statistical comparison between effect of small molecule and AITC controls. *p-value, statistical comparison between effect of small molecule and No AITC controls (2-way anova post hoc comparison).

**Table 2:** Baseline Velocity at 28.5°C. .

**Drug**	**Normalized Velocity**	**SEM**
1% DMSO	1.00	0.12
Buprenorphine	1.38	0.27
Clonidine	0.76	0.11
Hc-030031	0.73	0.12
Amitriptyline	0.89	0.10
TC-N 1752	1.01	0.11
A-803467	1.56	0.22
Gabapentin	1.00	0.10
Ibuprofen	0.80	0.12

Effect of small molecules of baseline velocity. Small molecule analgesics that did suppress larval velocity below that of No AITC controls in the AITC sensitized thermal aversion assay had no effect on baseline larval velocity. The small molecules tricaine (anesthetic) and pancuronium bromide (paralytic), which did significantly suppress larval velocity below that of No AITC controls, significantly inhibited baseline larval velocity indicating a non-analgesic effect on locomotion. p-value represents comparison between 1% DMSO and small molecule condition.

### Analgesic compounds reverse sensitized thermal aversion

To further test whether the AITC sensitized thermal aversion assay might be useful as a screening method for the identification of presumptive analgesics, we tested seven additional compounds with well characterized analgesic properties that have different molecular targets: the α2-adrenergic receptor agonist clonidine, the TRPA1 antagonist HC-030031, the tricyclic serotonin and norepinephrine transport inhibitor amitriptyline, the NaV_1.7_ antagonist TC-N 1752, the NaV_1.8_ antagonist A803467, the α2δ-ligand gabapentin, and the nonsteroidal anti-inflammatory drugs ibuprofen. As only a single concentration was used for each compound, a negative result is not an indication that a compound does not have analgesic properties.

The selective α2-adrenergic receptor agonist clonidine, originally identified for use as an antihypertensive agent, has subsequently been used in pain management [25, 26]. Clonidine is proposed to exert its analgesic effects by acting on both peripheral and central pain mechanisms, though on which pathways clonidine acts remains unclear [27]. Like buprenorphine, clonidine blunted AITC induced sensitized heat aversion, reducing place avoidance and velocity in the 31.5°C zone to levels observed in vehicle/no AITC-treated larvae (Fig. 4A, Table 1).

Since AITC acts through its receptor TRPA1 to induce nociception and neurogenic inflammation [18], we reasoned that a TRPA1 antagonist (HC-030031) would likely suppress AITC sensitized temperature discrimination [28–30]. Indeed HC-030031 dramatically reduced sensitized thermal aversion (Fig. 4A). HC-030031 also suppressed AITC induced increases in velocity in the 31.5°C zone to levels equivalent to those observed for vehicle/no AITC controls (Table 1). The tricyclic serotonin and norepinephrine transport inhibitor amitriptyline has been shown to be an effective analgesic for the treatment of inflammatory and neuropathic hyperalgesia, while having no effect on acute nociception [31–33]. Amitriptyline suppressed AITC sensitized aversion at 31.5°C and suppressed changes in velocity caused by AITC treatment (Fig. 4A, Table 1).

NaV_1.7_ is a voltage gated sodium channel that is specifically expressed in nociceptive sensory neurons [34–36]. Loss of function mutations in this gene have been shown to cause insensitivity to pain whereas gain of function mutations cause chronic pain conditions [37, 38]. It is therefore appears to be an excellent candidate for analgesic intervention. Indeed the small molecule antagonists including TC-N 1752 and antibodies against NaV_1.7_ have been shown to inhibit nociceptive behavior in rodent models of pain [39–43]. The zebrafish ortholog of NaV_1.7_, Scn1Laa is specifically expressed in somatosensory neurons [44]. TC-N 1752 significantly reduced the sensitizing effects of AITC but did not significantly suppress the elevated velocity induced by ATIC in the 31.5°C zone (Fig. 4A, Table 1).

Three of compounds that act as analgesics in other systems had no effect on AITC sensitized thermal avoidance. For example the voltage gated sodium channel NaV_1.8_ antagonist A-803467, which has potent anti-nociceptive properties in rodents, had no effect on sensitized avoidance but did reduce velocity to control levels [45] (Fig. 4A, Table 1). However, there does not appear to be a direct ortholog of NaV_1.8_ in zebrafish [44], most likely explaining why we observed no effect of this drug on place aversion. We also found no differences in thermal avoidance nor in velocity in the experimental zone between control and gabapentin treated larvae (Fig. 4A, Table 1). Gabapentin, a ligand for the α2δnon-essential subunit of L-type calcium channels, is closely associated with the treatment of neuropathic pain and has been found to be ineffective as a treatment for acute or inflammatory nociception in a number of studies [46, 47].

Lastly we tested whether or not the NSAID ibuprofen altered ATIC sensitized thermal aversion. Ibuprofen inhibits the production of the prostaglandins and NSAIDs are commonly used in the treatment of inflammatory pain [48]. NSAIDs have been reported to inhibit temperature hyperalgesia by acting on both central and peripheral pathways [49]. Additionally, ibuprofen has been shown to reduce the inflammatory response evoked by AITC [30]. Ibuprofen no effect on sensitized temperature avoidance and velocity (Fig. 4A, Table 1).

We also tested whether compounds that are not classified as analgesics would affect AITC sensitized thermal avoidance. In addition to naloxone, which did not effect sensitized thermal avoidance, we tested 1) the selective β_1_ adrenoreceptor antagonist, atenolol, commonly used in the treatment of hypertension, 2) the anticonvulsant, lamotrigine, used in the treatment of epilepsy and bipolar disorder, 3) the angiotensin-converting enzyme inhibitor, enalapril, used in the treatment of high-blood pressure, 4) the antihistamine, diphenhydramine, used in the treatment of allergies, 5) The proton-pump inhibitor, omeprazole, commonly uses in the treatment of gastroesophageal reflux disease and 6) promethazine, a phenothiazine derivative with antihistamine, anti nausea and sedative properties. None of these compounds affected AITC sensitized thermal aversion, though both atenolol and lamotrigine caused a reduction in velocity in the 31.5°C zone potentially pointing to an effect on locomotion by these drugs (Fig. 4B, Table 1).

We next assessed the effects of an anesthetic, tricaine, and a paralytic compound, pancuronium bromide. These compounds would be expected to cause sensory and/or motor impairment and therefore potentially inhibit thermal aversion. Tricaine completely abolished thermal aversion while pancuronium bromide reduced thermal aversion to levels induced by acute thermal aversion at 31.5°C (Fig. 4B). However both compounds significantly reduced velocity in the 31.5°C zone below levels observed for larvae in the acute thermal aversion assay, while none of the analgesic compounds effective in inhibiting AITC sensitized aversion suppressed velocity below that seen for vehicle only/No AITC treated controls (Table 1).

To further investigate if any of the compounds that reversed temperature aversion were also impeding motor function, we assayed the effects of each of the compounds on locomotion at rearing temperature. None of the analgesic compounds impaired velocity while tricaine and pancuronium bromide significantly reduced velocity at 28.5°C (Table 2). This provides evidence that the sensitized thermal aversion assay can distinguish between compounds that have analgesic properties versus those that suppress locomotion independent of an analgesic effect.

**Figure 5:**
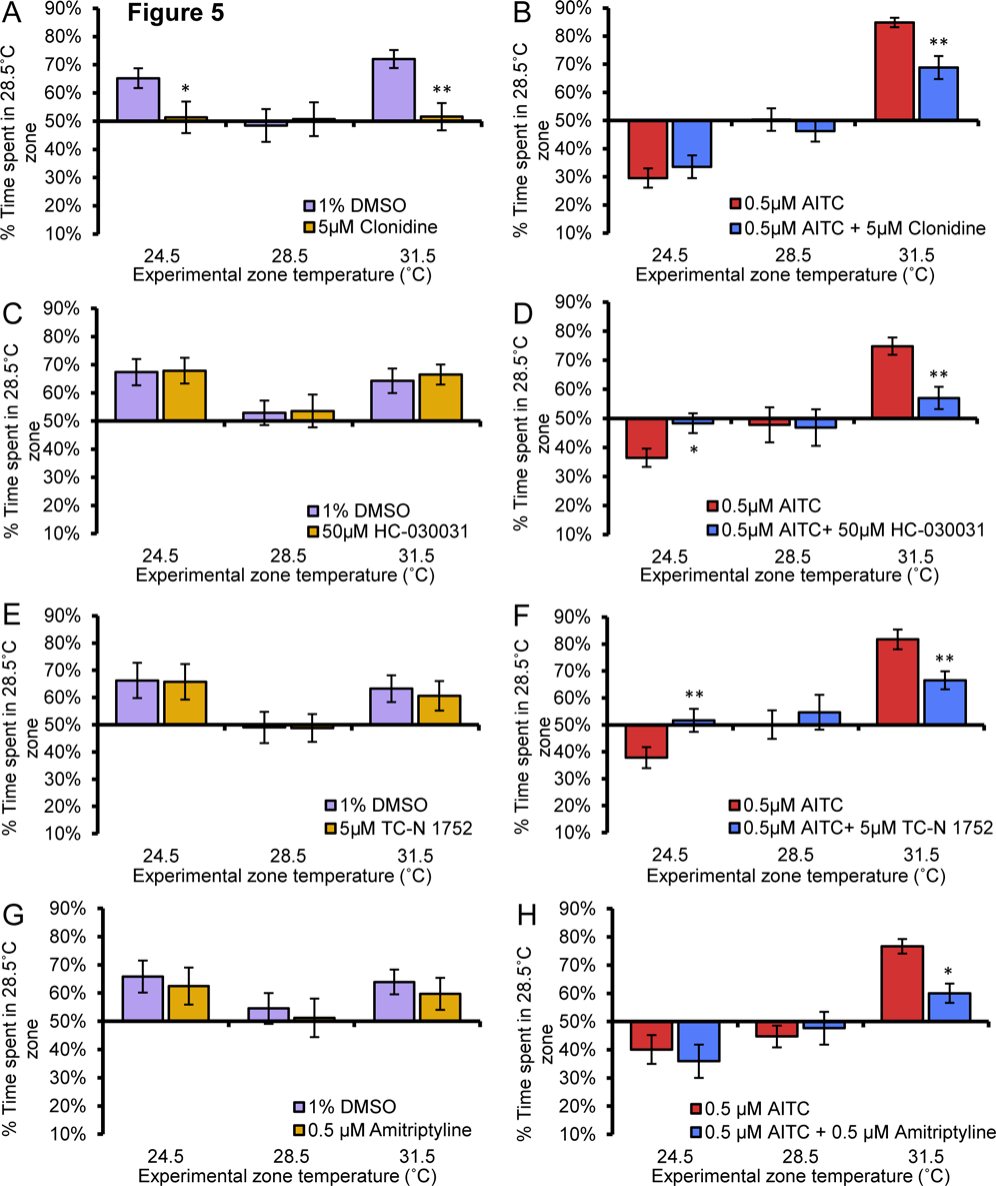
Analgesic molecules have differential effects on thermal aversion. **A**, Clonidine (5μM) significantly reduced 24.5°C and 31.5°C aversion when compared to vehicle treated larvae (2-way anova post hoc comparison) and eliminated aversion to 24.5°C and 31.5°C as measured by no significant difference from chance (1-way anova against a hypothetical mean of 50%). Clonidine did not induce aberrant zone preference (1-way anova against a hypothetical mean of 50%) and clonidine treated larvae did not differ from vehicle treated larvae in the 28.5°C vs 28.5°C assay (2-way anova post hoc comparison). **B**, Clonidine significantly reduced AITC sensitized aversion to 31.5°C but not 24.5°C preference when compared to vehicle treated larvae (2-way anova post hoc comparison). Clonidine did not induce aberrant zone preference (1-way anova against a hypothetical mean of 50%) and did not differ from vehicle treated larvae in the AITC sensitized 28.5°C vs 28.5°C assay. **C**, HC-030031 (50μM) treated larvae did not differ from vehicle-treated larvae in avoidance of either 24.5°C or 31.5°C zones (p>0.05, p>0.05, respectively, 2-way anova post hoc comparison. **D**, HC-030031 significantly reduced AITC sensitized aversion to 24.5°C and 31.5°C when compared to vehicle treated larvae (p<0.0X, p<0.01, respectively, 2-way anova post hoc comparison). HC-030031 did not induce aberrant zone preference (p>0.05, 1-way anova against a hypothetical mean of 50%) and did not differ from vehicle treated larvae in the AITC sensitized 28.5°C vs 28.5°C assay. **E**, TC-N 1752 (5μM) treated larvae did not differ from vehicle-treated larvae in avoidance of either 24.5°C or 31.5°C zones. **F**, TC-N 1752 significantly reduced AITC sensitized aversion to 24.5°C and 31.5°C when compared to vehicle treated larvae. TC-N 1752 did not induce aberrant zone preference (p>0.05, 1-way anova against a hypothetical mean of 50%) and did not differ from vehicle treated larvae in the AITC sensitized 28.5°C vs 28.5°C assay. **G**, Amitriptyline (0.5μM) treated larvae did not differ from vehicle-treated larvae in avoidance of either 24.5°C or 31.5°C zones (p>0.05, p>0.05, respectively, 2-way anova post hoc comparison). **H**, Amitriptyline significantly reduced AITC sensitized aversion to 31.5°C but not 24.5°C when compared to vehicle treated larvae (p<0.05, p>0.05 respectively, 2-way anova post hoc comparison). Amitriptyline did not induce aberrant zone preference (p>0.05, 1-way anova against a hypothetical mean of 50%) and did not differ from vehicle treated larvae in the AITC sensitized 28.5°C vs 28.5°C assay (p>0.05, 2-way anova post hoc comparison). * p<0.05, ** p<0.01, *** p<0.001. Error bars represent SEM.

Since clonidine, HC-030031, TC-N1752, and amitriptyline showed efficacy in the AITC sensitized thermal aversion assay, we tested all these compounds on our remaining temperature avoidance assays. Like buprenorphine, clonidine blocked acute hot and cold aversion (Fig. 5A). Clonidine blunted AITC induced sensitized heat aversion, reducing choice to similar levels observed in vehicle-treated larvae but had little effect on AITC-induced sensitized cool preference (Fig. 5B). Unlike buprenorphine and clonidine, HC-030031, TC-N1752 nor amitriptyline had any effect on acute hot or cold thermal aversion (Fig. 5C, 5E, 5G). HC-030031 and TC-N 1752 in addition to reversing AITC sensitized thermal aversion also inhibited AITC sensitized cool preference (Fig. 5D, 5F). Amitriptyline on the other hand had no effect on AITC sensitized cool preference (Fig. 5H).

## Discussion

Nociception occurs when intense chemical, temperature or mechanical stimulation is detected by diverse subpopulations (nociceptors) of peripheral neurons that arise in the trigeminal ganglion that innervates the head and the dorsal root ganglia that innervate the body or trunk. Previous studies have identified multiple subtypes of nociceptors in zebrafish suggesting a similar organization of nociceptive circuits between mammals and zebrafish even at timepoints as early as 1–3 dpf, [8, 50, 51]. Furthermore, many of the molecular receptors for nociception such as TRPV1 (noxious heat, pH, chemical) and TRPA1 (noxious chemosensation, including response to AITC) have conserved function in the zebrafish [8, 11].

We have established that individually arrayed 5 dpf zebrafish larvae make consistent choices when presented with two zones of different temperatures. They avoid temperatures deviating as little as 3°C above and 4°C below the rearing temperature of 28.5°C, with generally stronger aversion as the difference in temperatures widened. This behavior corresponds with our previous findings that trigeminal somatosensory neurons expressing the noxious heat receptor TRPV1 are activated with increasing intensity at temperatures greater than or equal to 28°C and that activation of TRPV1 drives thermal aversion behavior in zebrafish (Gau et al., 2013).

Thermal hyperalgesia is a common symptom of individuals suffering from chronic inflammatory pain [52]. Inflammatory compounds have been shown to shift and potentiate the thermal aversion of rodents [15, 53]. To model this process in zebrafish, we incubated larvae with the noxious inflammatory agent, AITC to induce neurogenic inflammation and inflammatory pain and has been shown to induce thermal hyperalgesia in a TRPV1 dependent manner [21]. We found that AITC incubation sensitized aversion to hot temperatures (≥31.5°C) and caused larvae to avoid rearing temperature (28.5°C) in favor of cooler temperatures that would otherwise be acutely aversive.

In addition to place aversion, we measured the velocities of larval movements in each zone, as increased velocity has previously been used as a measure of nociception in zebrafish [8, 11]. We found that velocity of the larvae corresponded with place aversion when warmer temperatures were analyzed, with increased velocity in the warm zone, but not when cooler temperatures were presented. After AITC sensitization velocity was consistently elevated in the warmer zone. For instance an elevation in velocity was seen in the 28.5°C zone after AITC treatment, but only when presented with a cooler temperature alternative. This result suggests that in this assay, nociceptive stimuli induce a greater behavioral response when juxtaposed with a less noxious environment and might reveal nociception that would be otherwise masked in a uniform environment.

A hallmark of nociception assays is that analgesic compounds should reverse the behavior in a dose dependent manner [3]. We hypothesized that that if our temperature aversion assay was modeling nociception, analgesic compounds should be able to blunt or inhibit aversion. To test this idea, we measured the efficacy of the opioid buprenorphine to disrupt temperature aversion in either acute or AITC-sensitized assays [22]. We found that buprenorphine was effective in suppressing temperature aversion in both the acute and AITC sensitized temperature aversion assays. Furthermore when measured using the AITC sensitized thermal aversion assay, buprenorphine was able to inhibit thermal aversion in a dose dependent manner. We were also able demonstrate that buprenorphine was acting via opioid receptors as the opioid receptor antagonist naloxone was able to reverse the action of buprenorphine on thermal aversion as has been shown for mammalian models of nociception using these drugs [54]. Based on these findings, we believe that we are likely modeling nociceptive behavior in our temperature aversion assays.

Because of the scalability of our assays and their relatively low cost, we reasoned that it might open up an avenue for large-scale high throughput screening for small molecule analgesics that are not practical to conduct in other model systems. By using our model where drugs are administered at an organismal level, one might be able to screen for molecules that can act anywhere throughout the highly complex and still not well understood nociceptive pathway(s). To see if a screen might be effective in identifying potential analgesics, we screened a number of analgesics and non-analgesics using the AITC sensitized thermal avoidance assay as this behavior was very robust and could be modeling complex inflammatory nociception. Only a single concentration of each compound was assessed usually greater than ten times the reported EC_50_ but also within the micromolar range utilized in many small molecule screens. Therefore failure for a molecule to inhibit temperature aversion should not be interpreted as a lack of analgesic properties. This is often the case in any screen where the goal is to identify potential candidates not every candidate. Of the eight analgesic compounds tested, five of the compounds, buprenorphine, clonidine, HC-030031, TC-N1752 and amitriptyline all reversed AITC sensitized thermal aversion and velocity to levels indistinguishable from vehicle-only (no AITC) treated larvae at the tested temperatures. This would suggest that these compounds were reducing sensitized aversion without effecting baseline aversion to heat in this assay as the animals still avoided the 31.5°C zone to levels equivalent with vehicle alone/no AITC treated larvae. The three others, gabapentin, ibuprofen and A-803467 had no effect in this assay. As mentioned this could be due to improper dosage or lack of efficacy in this assay due to a number of reasons. It may be that the molecular targets of these drugs do not exist in zebrafish as is the case for A-803467 or are significantly different in how they would interact with drugs. For example, capsaicin causes a burning sensation in mammals by interacting with TRPV1, but has no effect on zebrafish as the zebrafish TRPV1 does not bind and is insensitive to this compound (Gau et al., 2013). Another possibility is that the behavior being utilized is not a target for the tested compound. A number of analgesics are only effective in the treatment of specific pain conditions. It appears though that the AITC sensitized aversion assay may be a useful tool to identify analgesic molecules.

We next tested a number of therapeutics that are not widely characterized or used as analgesics. None of these compounds affected AITC sensitized thermal aversion, suggesting that a de novo screen might not have a high false positive rate. Three of the compounds that did not affect sensitized thermal aversion, A-803467, atenolol and lamotrigine did inhibit velocity during testing, suggesting that is possible to suppress locomotion without affecting place aversion. It is possible that these compounds would have scored as positives in a purely locomotion based nociception assay and provides evidence that the place aversion based assay is more selective than a solely locomotor based assay.

To test if we could identify compounds that might impair place aversion by suppressing motor function, we assayed two compounds the anesthetic, tricaine and the paralytic, pancuronium bromide. Both of these compounds blocked sensitized thermal avoidance, but they also significantly reduced velocity below that seen for vehicle only/no AITC treated larvae. It might be prudent then to exclude molecules that suppress locomotion beyond that seen for vehicle only/no AITC controls or to perform a secondary screen on all compounds found to diminish place aversion for effects on motor function. When all the compounds that reversed thermal aversion were tested for effects on baseline locomotion only tricaine and pancuronium bromide suppressed larval velocity. This would indicate that velocity measurements taken during the AITC sensitized thermal avoidance assay could be predictive of molecules that would suppress locomotion independently of analgesic properties.

Through additional secondary screening with acute hot and cold temperature aversion assays and AITC sensitized cool preference assays, it should be possible to further classify the properties of small molecules identified as being effective in the AITC sensitized thermal aversion assay. Our initial characterization of analgesics that suppressed AITC sensitized thermal aversion revealed differential effects on the various assays. For instance some compounds at the concentration tested inhibited sensitized temperature aversion without having any effect on acute temperature aversion. The effectiveness of a potential analgesic in each of these assays could be informative in determining its therapeutic potential.

A behavioral phenotype based small molecule screen offers a separate pathway from traditional candidate target based approaches. Indeed phenotypic observations have been very successful in identifying analgesics, often as off-target effects of drugs used for other purposes. While nociception screens in model systems such as *C. elegans* and *Drosophila* have been somewhat successful, modeling nociceptive behavior in these models systems is limited by the fundamental differences between their nervous systems and those of vertebrates [55–57]. Though zebrafish have been used to examine nociception previously, the main criterion has been velocity changes in a chamber, and drugs that would inhibit motor behavior would be identified as false positives. By testing behavioral discrimination and velocity concurrently, these confounds would be eliminated.

Based on our findings, we believe it is possible to model nociception in zebrafish larvae and that the assays we have developed could provide a way forward for analgesic discovery that may offer a complementary parallel pathway to target-based drug development. Furthermore advances in measuring the neuronal activity at single neuron resolution in the entire zebrafish larval nervous system, provides the opportunity to discover on which neurons in the pain pathway a potential analgesic is acting on [58]. This would allow further characterization of potential analgesic before testing in other animal models. We also believe the place preference assay can be adapted through the expression of channel rhodopsin in specific neuronal circuits to determine the attractive or aversive nature of any neuronal circuit in the zebrafish and thereby open up opportunities for therapeutic discovery for a variety of neurological diseases.
